# Search of Reflectance Indices for Estimating Photosynthetic Activity of Wheat Plants Under Drought Stress

**DOI:** 10.3390/plants14010091

**Published:** 2024-12-31

**Authors:** Firuz Abdullaev, Daria Churikova, Polina Pirogova, Maxim Lysov, Vladimir Vodeneev, Oksana Sherstneva

**Affiliations:** 1 Department of Biophysics, National Research Lobachevsky, State University of Nizhny Novgorod, 23 Gagarin Avenue, 603022 Nizhny Novgorod, Russia; 2 N.I. Vavilov All-Russian Institute of Plant Genetic Resources (VIR), 42–44 Bolshaya Morskaya, 190000 Saint Petersburg, Russia; 3 Department of High-Performance Computing and System Programming, National Research Lobachevsky State University of Nizhny Novgorod, 23 Gagarin Avenue, 603950 Nizhny Novgorod, Russia

**Keywords:** hyperspectral imaging, PAM imaging, photosynthesis, chlorophyll fluorescence, abiotic stress, drought stress, tolerance, *Triticum aestivum* L.

## Abstract

Global climate change and the associated increasing impact of droughts on crops challenges researchers to rapidly assess plant health on a large scale. Photosynthetic activity is one of the key physiological parameters related to future crop yield. The present study focuses on the search for reflectance parameters for rapid screening of wheat genotypes with respect to photosynthetic activity under drought conditions. The development of drought stress modelled in laboratory conditions by stopping irrigation caused changes in chlorophyll fluorescence parameters that corresponded to a decrease in photosynthetic activity. In particular, a decrease in the photochemical quantum yield of photosystem II (Φ_PSII_), which characterizes the rate of linear electron transport in the photosynthetic electron transport chain and is one of the most sensitive parameters responding at the early stages of drought stress, was observed. Along with the measurement of the photosynthetic activity, spectral characteristics of wheat plants were recorded using hyperspectral imaging. Normalized difference indices (NDIs) were calculated using the reflectance intensity of wheat shoots in the range from 400 to 1000 nm. Four NDIs that showed a strong correlation with the level of photosynthetic activity estimated by Φ_PSII_ were selected from different wavelength ranges (NDI_610/450_, NDI_572/545_, NDI_740/700_, and NDI_820/630_). The indices NDI_572/545_ and NDI_820/630_ showed the best combination of sensitivity to soil moisture deficit and strong relationship with photosynthetic activity under drought stress. Possible molecular and physiological causes of this relationship are discussed. The use of the proposed indices will allow to monitor in detail the specific features of wheat plant response and can serve as one of the criteria for selection of the most promising genotypes in breeding of drought-tolerant cultivars.

## 1. Introduction

Wheat is an important crop that provides a significant portion of the calories and protein consumed by humans [1,2]. The efforts of breeders have resulted in cultivars that produce high yields under favorable growing conditions. Yield losses are strongly related to stress factors, among which drought is one of the most devastating. According to [3], drought-induced yield losses can reach up to 15%. These losses have been increasing in recent years, including due to the climate change [4]. In addition, the negative effect of soil moisture deficit can be enhanced by soil salinity [5,6,7]. The problems outlined above challenge breeders to obtain new cultivars that give high yields under drought stress.

Yield losses under drought stress are associated with a complex of changes, including a decrease in the activity of photosynthesis, which is a key component of the production process. The crucial role of photosynthesis in productivity has led researchers to pay great attention to the study of its activity under drought conditions. The results of such studies are summarized in reviews [8,9,10,11]. The following general patterns of changes in photosynthetic activity during drought progression can be identified. The early stage of drought-induced changes in the activity of the photosynthetic apparatus (PSA) is associated with modulation of stomatal conductance, reduction in CO_2_ availability, and suppression of linear electron transport in the photosynthetic electron transport chain (PETC) due to a slowdown of light-independent reactions and enhancement of the non-photochemical quenching of fluorescence (NPQ) mechanism [12,13]. These processes are accompanied by a decrease in chlorophyll fluorescence (ChlF) parameters such as the effective photochemical quantum yield of PSII (Φ_PSII_), the coefficient of photochemical fluorescence quenching (qP), and the electron transport rate (ETR) and an increase in NPQ, the quantum yield of regulated non-photochemical energy dissipation in PSII (Φ_NPQ_), and the coefficient of non-photochemical fluorescence quenching (qN), etc. [14]. Long-term drought results in damage to photosynthetic proteins and thylakoid membranes caused by reactive oxygen species (ROS), the increased accumulation of which accompanies the early stage [15]. Such destructive changes are detected together with a decrease in the maximum quantum efficiency of PSII (F_v_/F_m_) and, in some cases, a decrease in NPQ [14].

The development of drought-tolerant cultivars is based on genotyping and phenotyping [14,16]. Genomic approaches have identified a number of genotypic traits associated with drought tolerance [17,18]. In addition, instrumental phenotyping, including remote optical methods, has been actively developed in recent years [19,20]. Screening of genotypes with respect to higher photosynthetic potential and stability under water deficit conditions is performed. In particular, the method of ChlF registration demonstrates high information content [14]. Its successful application for phenotyping in breeding under both laboratory and field conditions has been demonstrated. Despite the advantages of the ChlF method, including the ability to directly record photosynthetic activity, it has a throughput limitation due to the duration of an individual measurement. This has led to a search for other approaches that can correctly assess drought stress-induced alterations in photosynthetic activity. These include, first of all, spectral imaging methods, which make it possible to carry out larger-scale studies due to high speed and large scale of measurements. A number of studies in recent years have shown that changes in the reflectance spectrum can be associated with changes in photosynthesis activity [21,22,23,24].

Various indices calculated using the values of the reflection coefficients at two or more wavelengths are widely used to assess the state of plants. Among them, the structural, water, and xanthophyll indices are the most commonly correlated with plant water stress [25]. Structural indices are based on measurements in the visible (VIS) and near-infrared (NIR) range. These include the normalized difference vegetation index (NDVI), the renormalized difference vegetation index (RDVI), optimized soil-adjusted vegetation index (OSAVI), and others. In addition to using structural indices to assess yields, they are used to monitor plant stress, in particular, to detect changes caused by water shortage [26,27]. The next group includes the water indices, measured in NIR: water index (WI), simple ration water index (SRWI), normalized difference water index (NDWI), and others. This group of indices makes it possible to estimate the water content in plants. Another subgroup, xanthophyll indices, includes the photochemical reflectance index (PRI) and its various modifications [25]. The PRI is sensitive to the epoxidation state of the xanthophyll cycle pigments, and is also related to NPQ and photosynthesis efficiency [23].

Despite active research in this area, a comprehensive body of knowledge sufficient to recommend a specific approach for assessing the photosynthetic response to drought in individual genotypes using spectral imaging is currently lacking.

The aim of this work was to identify reflectance indices that can act as indicators of drought-induced changes in photosynthesis activity. We hypothesized that drought-induced shifts in a number of wheat reflectance indices caused by alterations in leaf structure and pigment composition may be associated with the changes in photosynthetic activity. Identification of such indices will make it possible to monitor in detail the peculiarities of the response of individual genotypes in breeding trials. This, in turn, will make it possible to select drought-tolerant genotypes as well as to identify genetic markers for genomic selection that reflect a specific pattern of the response to drought.

## 2. Results

### 2.1. Effect of Soil Moisture Deficit on Phenotypic Parameters of Wheat Seedlings

#### 2.1.1. Morphometric Parameters

**Figure 1:**
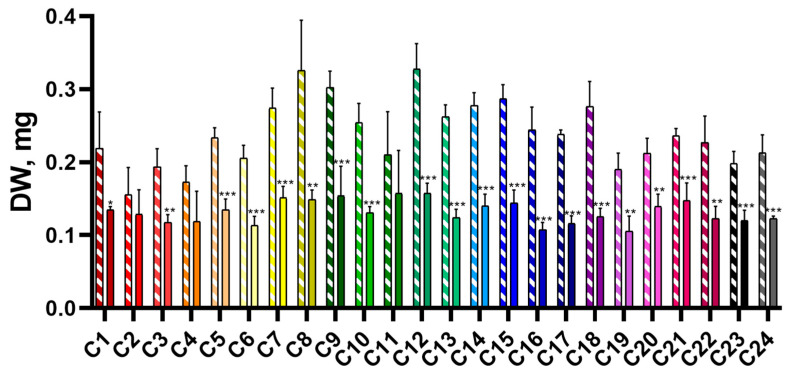
Dry weight of 28-day-old wheat seedlings of the CC (shaded bars) and DS (solid bars) groups. Data are presented as mean ± SEM. *, **, *** indicate significant differences between experimental and control groups (*t*-test, * *p* < 0.05; ** *p* < 0.01; *** *p* < 0.001).

A comparison of biomass accumulation and the water content (WC) in control plants (CC) and plants subjected to drought stress (DS) was performed to evaluate the effect of short-term soil drought on morphometric parameters of seedlings. It was shown that dry weight (DW) of CC plants ranged from 155 ± 19 mg (C2) to 327 ± 17 mg (C12) (Figure 1). DW of DS plants was significantly lower than the control plants and ranged from 105 ± 11 mg (C19) to 157 ± 7 mg (C12).

**Figure 2:**
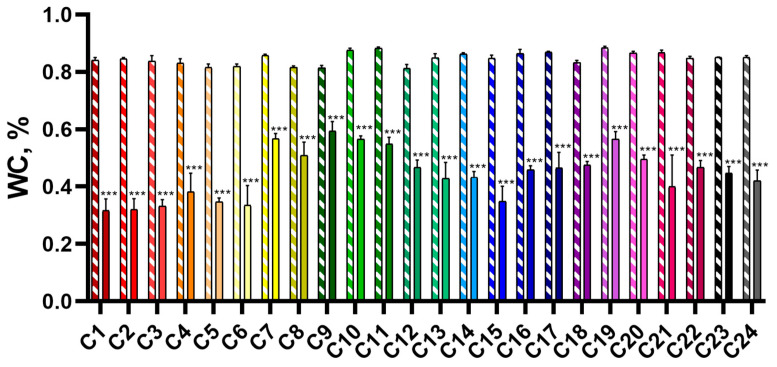
WC in 28-day-old wheat seedlings of the CC (shaded bars) and DS (solid bars) groups. Data are presented as mean ± SEM. *** indicates significant differences between experimental and control groups (*t*-test, *p* < 0.001).

WC in the CC group ranged from 81.3 ± 1% in C12 to 88.4 ± 0.3% in C19; in the DS group, WC ranged from 31.6 ± 2% in C1 to 59.3 ± 1.7% in C9 (Figure 2). On average for all cultivars, WC in plants of the DS group decreased by 40.2 ± 1.7% (from 22.0 ± 1.6% in C9 to 52.6 ± 1.8% in C2) compared to CC plants. Figure 2 shows the values of WC in 28-day-old plants of the CC and DS groups.

#### 2.1.2. Photosynthetic Activity

**Figure 3:**
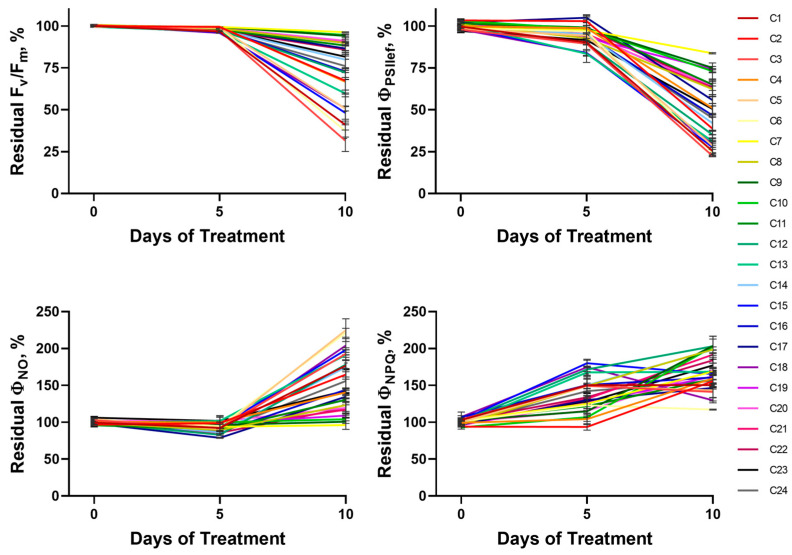
Dynamics of residual levels of ChlF parameters during drought development. Data are presented as mean ± SEM.

A 10-day soil drought caused dramatic changes in photosynthetic activity parameters based on changes in ChlF (Figure 3 and Figure S1). To investigate the effect of drought on photosynthetic activity parameters (F_v_/F_m_, Φ_PSII_, the quantum yield of non-regulated non-photochemical energy loss in PSII (Φ_NO_), and Φ_NPQ_) in wheat seedlings, we analyzed the dynamics of their residual level (as a percentage of control), including values before the onset of drought (at the age of 14 days), as well as after 5 and 10 days of DS (at the ages of 19 and 24 days, respectively) (Figure 3 and Figure S2). The normalization of the values of the experimental plants to the control plants allows us to level out the contribution of age-related alterations in photosynthetic activity to the patterns of drought-induced changes. The studied cultivars showed a variety of strategies for response to the induction and development of soil drought. In particular, four types of dynamics of photosynthesis activity parameters were revealed. The first group of cultivars showed a pronounced decrease in photosynthetic activity at the fifth day of DS, manifested in Φ_PSII_ decline and Φ_NPQ_ growth; this decrease intensified with the development of water stress by the 10th day of treatment. It is worth noting that the suppression of photosynthesis at the early stage of stress development (the fifth day of DS) was manifested only in the fall of Φ_PSII_ and rise of Φ_NPQ_; the pronounced changes of F_v_/F_m_ and Φ_NO_ were absent. This indicates that changes in the functioning of the PSA, not associated with damage to photosystem components, occurred at the fifth day of DS. Further drought development (10 days duration) was accompanied by significant changes in all studied parameters, including decreases in F_v_/F_m_ and Φ_PSII_ and increases in Φ_NO_ and Φ_NPQ_. The second group of cultivars showed a different pattern of changes: at the early stage of drought development (day 5), no pronounced changes in photosynthetic activity parameters were observed; at the same time, at the 10th day, a strong photosynthetic suppression comparable to that observed in the first group was registered. The third pattern of changes in ChlF parameters: early (day 5 of drought stress) statistically significant drop in photosynthetic activity and maintenance of moderate photosynthetic activity at the 10th day (the greatest level of photosynthetic activity compared to the previous groups). The fourth pattern: no significant change in photosynthetic activity at the early DS stage and its moderate decline at the late stage, comparable to the third group.

Summarizing the patterns of changes in the four photosynthetic parameters, the described groups of cultivars can be classified as: (i) cultivars with low PSA tolerance (NT); (ii) cultivars capable of maintaining stable PSA functioning under short-term water deficit but not tolerant to long-term intensive drought (MT); (iii) cultivars with high PSA tolerance to DS, showing reduced photosynthetic activity under moderate drought and no dramatic suppression of photosynthetic processes under severe drought (HT); (iv) cultivars with high photosynthetic tolerance to DS, maintaining stable functioning of PSA under both short-term and longer-term drought (HHT).

**Figure 4:**
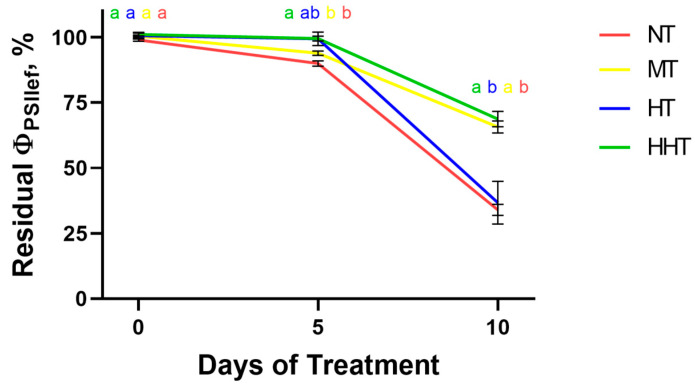
Drought-induced dynamics of residual Φ_PSII_ level of four groups of cultivars classified according to the patterns of changes in photosynthetic activity during drought development. Data are presented as mean ± SEM. NT—group with low photosynthetic tolerance, MT—group with high photosynthetic tolerance to the short-term moderate DS but not tolerant to long-term intensive DS, HT—group showing reduced photosynthetic activity under short-term moderate DS and high photosynthetic tolerance to the long-term intensive DS, HHT—group with high photosynthetic tolerance to both short-term and longer-term DS. Significant differences between the groups are indicated by different letters (ANOVA followed by Tukey’s test, *p* < 0.05).

All tested cultivars were divided into the four groups described above according to the dynamics of changes in Φ_PSII_, which is one of the key ChlF parameters reflecting the activity of photosynthetic processes: the NT group included cultivars C1, C3, C5, C12, C13, C14, C15, C16, C18, C23, and C24; MT—C2, C4, and C6; HT—C8, C19, C20, and C21; HHT—C7, C9, C10, C11, C17, and C22. The average dynamics of residual Φ_PSII_ for each group were obtained by summarizing data from the respective cultivars (Figure 4). The analysis of the obtained dynamics showed good agreement with the characterized patterns of drought-induced changes, including their qualitative and quantitative characteristics.

#### 2.1.3. Reflectance Parameters

**Figure 5:**
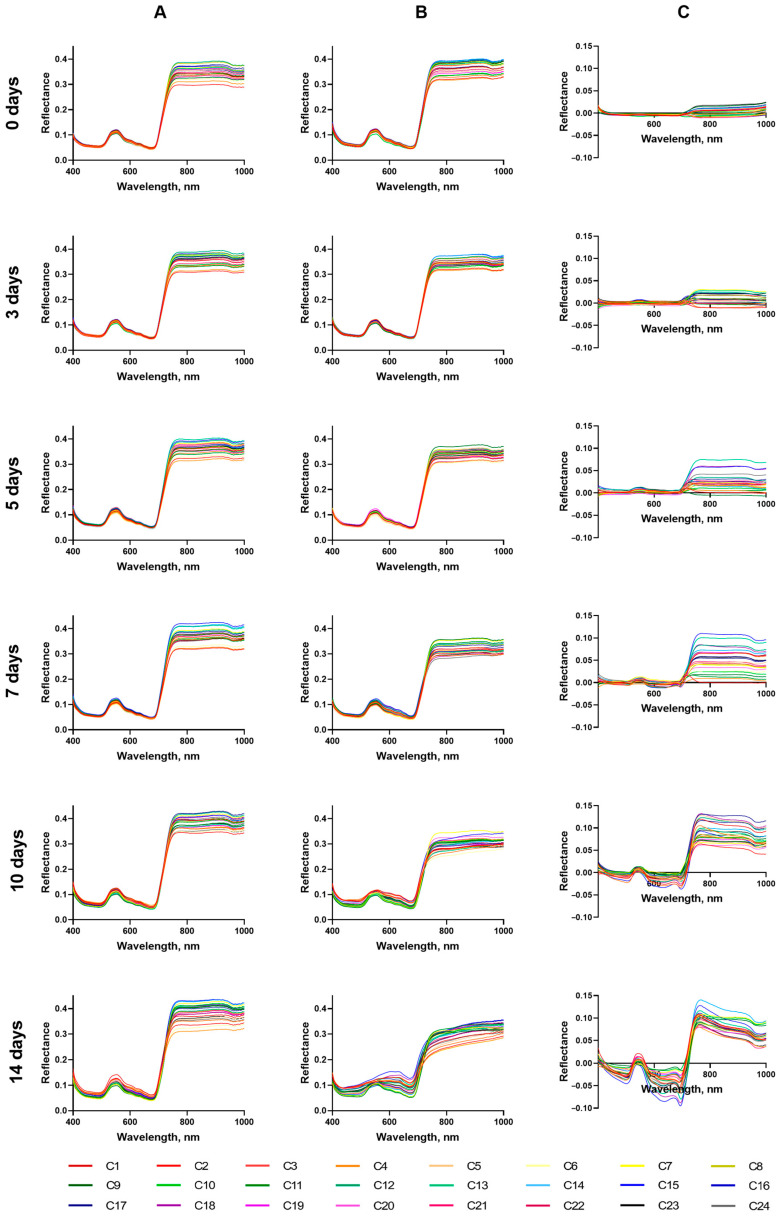
Reflectance spectra of CC (**A**) and DS (**B**) plants, and the difference spectra between CC and DS plants (**C**) at different days of drought. Data are presented as mean spectra for each cultivar.

Reflectance spectra of shoots before the start of the drought and 3, 5, 7, 10, and 14 days after the cessation of irrigation (at the ages of 14, 17, 19, 21, 24, and 28 days, respectively) were obtained by analyzing hyperspectral images of CC (Figure 5A) and DS plants (Figure 5B).

Water deficit in the soil significantly affected the optical properties of shoots, which was manifested in the change in the shape of reflectance spectra, progressing with the development of drought stress (Figure 5B). In the VIS region, loss of the spectrum shape typical for green parts of the plant due to optical properties of photosynthetic pigments, mainly absorption of light by chlorophyll, is observed. The sharp change in the reflection coefficient in the region of the red edge (RE), characteristic for green shoots of unstressed plants, loses clear boundaries and sharpness. The reflectance intensity in the region following the red edge (from 780 to 1000 nm, NIR) also decreases. Analysis of the difference between CC and DS spectra showed that the greatest changes in the reflectance spectra of plants were observed in the ranges from 550 to 700 nm and from 730 to 1000 nm (Figure 5C). It is worth noting that the greatest differences between the response of the reflectance parameters of different genotypes appear on 3–10 days of drought (age 17–24 days); with further development of drought, these differences decrease due to fatal for most plants structural and functional changes.

**Figure 6:**
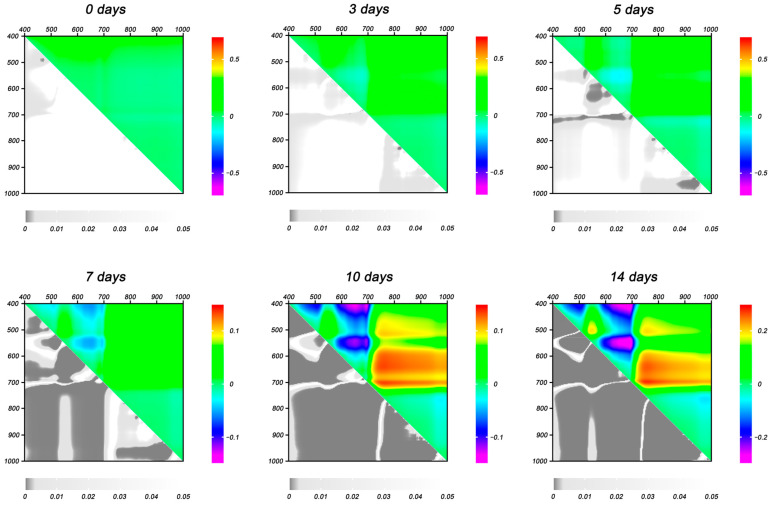
Heat maps of NDI differences between CC and DS plants (∆NDIs) at different days of drought. Data are presented as mean ∆NDIs (**top right**) and *p*-values (**bottom left**).

The effect of drought stress on normalized difference indices (NDIs) was assessed by analyzing heat maps of NDIs calculated at all studied wavelengths in the spectrum range from 400 to 1000 nm (Figure 6). The regions on heat maps (NDIs sets) that responded the earliest to changes in plants induced by soil moisture deficit were identified. In particular, statistically significant differences between NDIs of CC and DS plants were observed in the ranges: λ_1_ 510–600, λ_2_ 400–500; λ_1_ 490–660, λ_2_ 550–590; λ_1_ 700–720, λ_2_ 400–680; λ_1_ 810–1000, λ_2_ 780–800. An increase in the duration of drought stress was accompanied by an increase in the magnitude of the differences and their statistical significance (Figure 6). In addition, the areas on the NDI heat map showing a significant response to water deficit increased with the development of drought stress; starting from the seventh day of drought treatment and later, they covered almost the entire range studied.

### 2.2. Search for NDIs Reflecting Photosynthetic Activity Under Drought Stress

**Figure 7:**
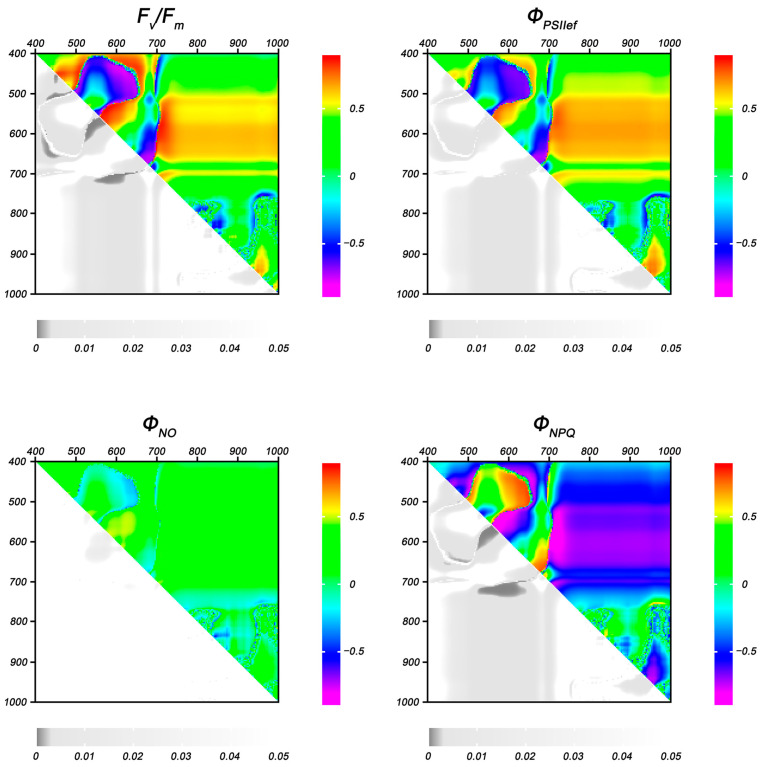
Heat maps of Pearson correlation coefficients between NDIs (in % of control) and the residual values of ChlF parameters in wheat plants after 5 days of DS. Data are presented as correlation coefficients (**top right**) and *p*-values (**bottom left**).

**Figure 8:**
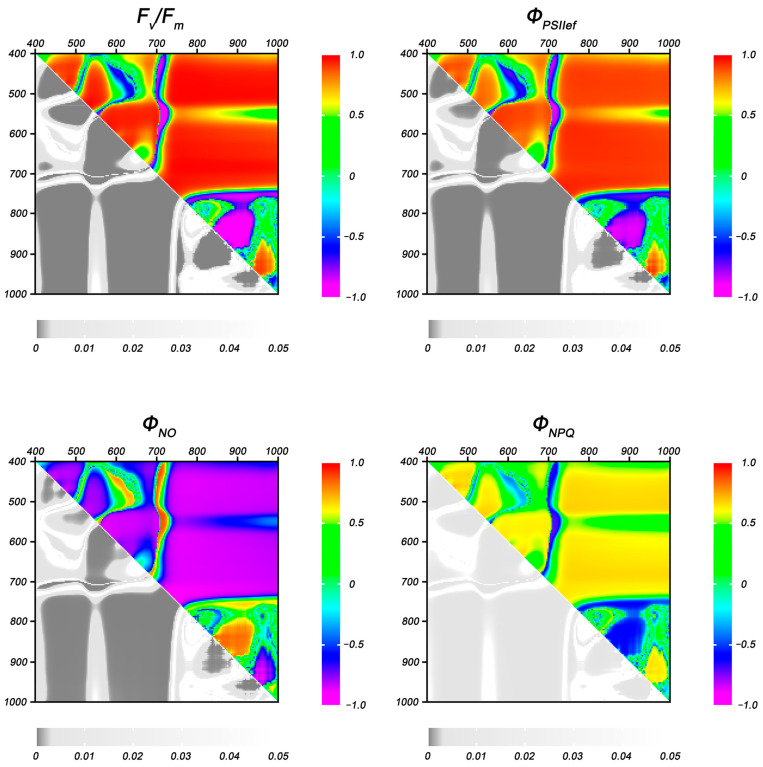
Heat maps of Pearson correlation coefficients between NDIs (in % of control) and the residual values of ChlF parameters in wheat plants after 10 days of DS. Data are presented as correlation coefficients (**top right**) and *p*-values (**bottom left**).

A correlation analysis of the relationship between the residual levels of ChlF parameters and the values of NDIs in DS plants as a percentage of control was carried out to identify NDIs capable of serving as indicators of photosynthesis suppression under drought stress. Heat maps of the correlation coefficients of the indicated parameters at the fifth and 10th day of stress treatment are presented in Figure 7 and Figure 8.

It was shown that the widest areas on the NDI heat maps with statistically significant correlation were shown for F_v_/F_m_, Φ_PSII_, and Φ_NPQ_ at the initial stage of drought (5 days of DS); the boundaries of these areas almost completely overlapped for the above ChlF parameters (Figure 7). The NDIs calculated at wavelengths λ_1_ 580–640, λ_2_ 440–500 nm showed the highest correlation coefficients (up to −0.77 for F_v_/F_m_, −0.69 for Φ_PSII_, and 0.80 for Φ_NPQ_); λ_1_ 570–600, λ_2_ 530–560 nm (up to 0.87 for F_v_/F_m_, 0.75 for Φ_PSII_, and −0.86 for Φ_NPQ_); λ_1_ 665–690, λ_2_ 580–660 nm (up to −0.74 for F_v_/F_m_, −0.75 for Φ_PSII_, and 0.76 for Φ_NPQ_); λ_1_ 700–720, λ_2_ 570–630 nm (to −0.85 for F_v_/F_m_, 0.77 for Φ_PSII_, and −0.87 for Φ_NPQ_). The residual level of Φ_NO_ showed a statistically significant correlation in only one range of NDIs (λ_1_ 575–640, λ_2_ 530–570 nm); the maximum value of the correlation coefficient was 0.50.

With the increase in the duration of drought stress, the regions with statistically significant correlation expanded and covered a major part of the heat maps; the distribution patterns of these regions and their boundaries were close for all ChlF parameters (Figure 8). On the other hand, one of the regions showing a fairly high correlation with the residual values of ChlF parameters on the fifth day of DS (λ_1_ 580–640, λ_2_ 440–500 nm) partially lost significant relationship by the 10th day; the highest correlation coefficients were observed in the ranges λ_1_ 585–610, λ_2_ 440–460 nm and λ_1_ 610–630, λ_2_ 460–490 nm (−0.70, 0.60, and −0.75 for F_v_/F_m_, Φ_PSII_, and Φ_NPQ_, respectively). There was no statistically significant correlation with the residual levels of Φ_NPQ_ in this range.

Given the distribution patterns of NDIs showing statistically significant correlation with residual ChlF parameters and the areas in the heat maps with the highest correlation coefficients, one NDI in each of the four areas described above was selected for further analysis: NDI_610/450_, NDI_572/545_, NDI_740/700_, and NDI_820/630_. The averaged drought-induced dynamics of these NDIs (in % of control) for each cultivar are shown in Figure S3.

**Figure 9:**
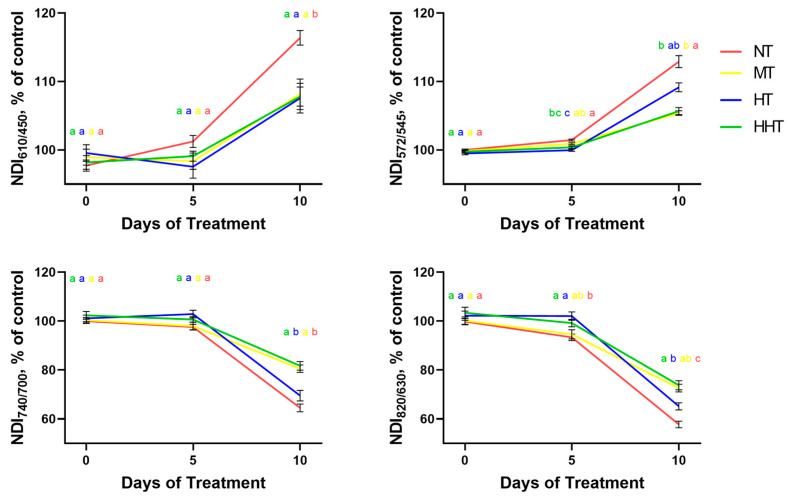
Drought-induced dynamics of NDIs (in % of control) of four groups of cultivars classified according to the patterns of changes in photosynthetic activity during drought development. Data are presented as mean ± SEM. NT—group with low photosynthetic tolerance, MT—group with high photosynthetic tolerance to the short-term moderate DS but not tolerant to long-term intensive DS, HT—group showing reduced photosynthetic activity under short-term moderate DS and high photosynthetic tolerance to the long-term intensive DS, HHT—group with high photosynthetic tolerance to both short-term and longer-term DS. Significant differences between the groups are indicated by different letters (ANOVA followed by Tukey’s test, *p* < 0.05).

At the next step, we analyzed the dynamics of NDI values of DS plants normalized to control values within the groups assigned according to the patterns of changes in photosynthetic activity (Figure 9). Analysis of the dynamics of changes in these NDIs showed that the greatest correspondence with the changes in current photosynthetic activity, estimated by the dynamics of the residual Φ_PSII_ level, was revealed for NDI_572/545_; there was a clear separation between groups, similar to that for residual Φ_PSII_. For the majority of indices, multiple comparisons of quantitative characteristics of different groups showed less clear separation than for Φ_PSII_. NDI_610/450_ performed worse than other analyzed indices: the MT, HT, and HHT groups did not differ significantly from each other either at the fifth or at the 10th day of DS. At the same time, the patterns of changes of the other NDIs (NDI_572/545_, NDI_740/700_, and NDI_820/630_) during drought development correspond well to those for Φ_PSII_.

**Figure 10:**
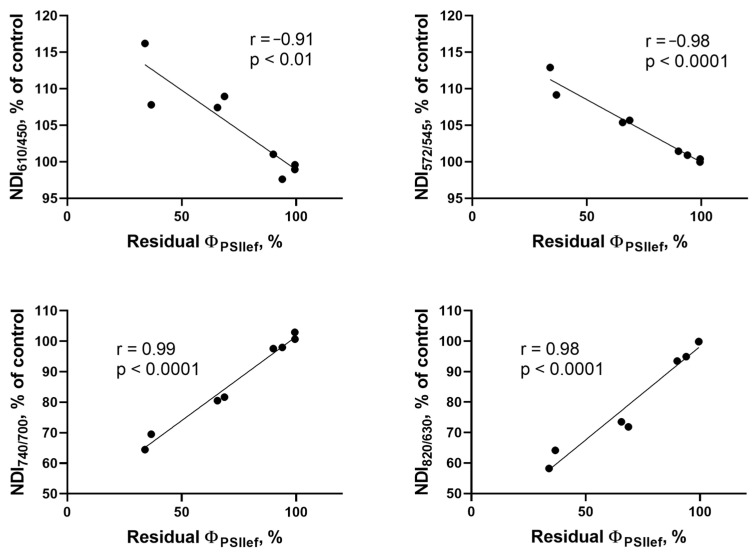
Correlation analysis of the relationship between NDIs (in % of control) and the residual value of Φ_PSII_ in four groups of cultivars classified according to the patterns of changes in photosynthetic activity during drought development (after 5 and 10 days of DS). The charts show Pearson linear coefficients and the *p*-values (two-tailed).

To quantitatively assess the applicability of DS plants’ NDIs normalized to control as indicators of the residual photosynthetic activity levels, correlation analyses of the relationships between these values were performed for the NT, MT, HT, and HHT groups on days 5 and 10 of drought (Figure 10). All NDIs showed high correlation with the residual Φ_PSII_; NDI_610/450_ showed the lowest degree of association with the residual Φ_PSII_ (r = −0.91, *p* < 0.01 and NDI_740/700_ showed the highest (r = 0.99, *p* < 0.0001).

**Figure 11:**
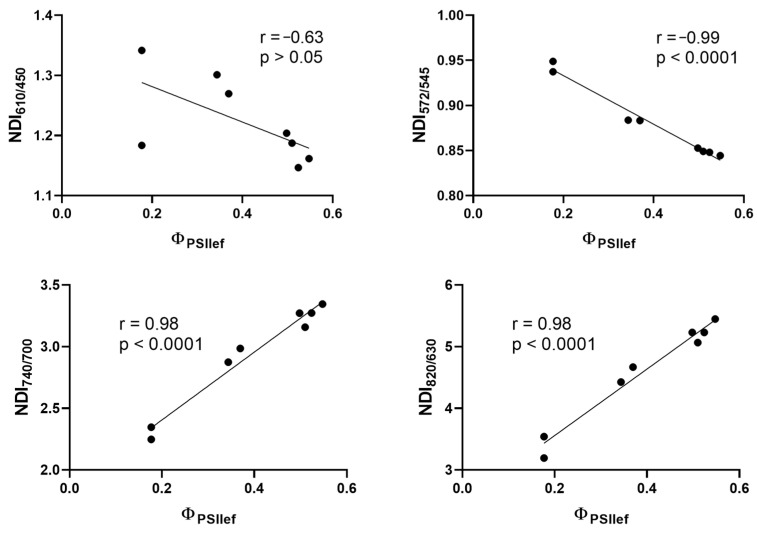
Correlation analysis of the relationship between the absolute values of NDIs and Φ_PSII_ in four groups of cultivars classified according to the patterns of changes in photosynthetic activity during drought development (after 5 and 10 days of DS). The charts show Pearson linear coefficients and the *p*-values (two-tailed).

The next step was to analyze the possibility of using absolute values of NDIs of stressed plants to directly assess the current level of photosynthetic activity under drought stress. This approach eliminates the need for a control group of plants to perform the analysis (Figure 11). Correlation analysis of the relationship between absolute values of NDIs and Φ_PSII_ of plants subjected to 5- and 10-day soil drought showed a strong statistically significant relationship of the studied values. The indices NDI_572/545_, NDI_740/700_, and NDI_820/630_ showed a high correlation with Φ_PSII_ in DS conditions; however, no statistically significant relationship was shown in the case of NDI_610/450_ (r = −0.63, *p* > 0.05).

## 3. Discussion

### 3.1. Effect of Drought Stress on Morphophysiological Characteristics of Wheat

Water deficit causes changes in a number of morphophysiological characteristics in plants, leading to dramatic consequences under prolonged and/or high-intensity stress [28,29]. This effect is most pronounced at the stages of plant development characterized by intensive growth of vegetative parts [30,31]. It is worth noting that the sensitivity of wheat plants to water deficit changes during ontogenesis; the most critical developmental stages for survival and final productivity are tillering, booting, and flowering [30,31,32,33]. The rapid vegetative growth stage, which requires high water use intensity, is one of the most limiting stages during drought stress [30]. In the present work, studies were carried out at the stage of intensive vegetative growth of wheat plants for which acute rapid soil drought was the cause of a decrease in water content and biomass (dry matter) accumulation; the magnitude of changes in morphometric parameters varied among different genotypes.

One of the main reasons for the decrease in biomass accumulation is the suppression of photosynthetic activity [34], which was recorded in this study under soil moisture deficit conditions in all the wheat cultivars studied. ChlF parameters changed at different stages of drought development. In particular, a decrease in Φ_PSII_ and an increase in Φ_NPQ_ were observed 5 days after stopping irrigation. A longer and more intensive 10-day drought resulted, along with the changes described, in a fall in F_v_/F_m_ and an increase in Φ_NO_. The directionality and dynamics of changes in ChlF parameters are consistent with the data described in other works [35,36,37,38,39,40,41]. A meta-analysis of the drought effects on wheat showed a decrease in Φ_PSII_ and F_v_/F_m_; and the decrease in F_v_/F_m_ was enhanced under more severe drought conditions [36]. The work [37] showed a decline in F_v_/F_m_ on the fourth and fifth day and an increase in NPQ on the third, fourth, and fifth day of acute drought. In [38], a multiphase development of drought was shown; the increase in NPQ (days 13–17 after irrigation was stopped) was followed by a decrease in F_v_/F_m_ and a greater decline in NPQ (days 18–24). It is worth noting that in most cases, as in our present study, ChlF parameters that characterize the rate of photosynthetic electron-transport chain activity (e.g., Φ_PSII_, NPQ, Φ_NPQ_, etc.) respond to drought stress earlier than parameters that characterize the functional integrity of the PSA (e.g., F_v_/F_m_) [42,43,44]. This effect is caused by the complex reaction of the plant to drought, one of the first stages of which is the closure of stomata and reduction in RuBisCO activity, which reduces the rate of the Calvin–Benson cycle and, as a consequence, causes suppression of linear electron transport in the PETC. In this case, a decrease in Φ_PSII_, qP, and ETR and an increase in NPQ, Φ_NPQ_, qN, etc., are observed. The increased production of ROS accompanying these events leads to damage to photosynthetic proteins and thylakoid membranes. Such changes significantly contribute to photosynthetic activity under intense and/or long-term drought stress and are detected together with a decrease in F_v_/F_m_ and, in some cases, a decrease in NPQ [14].

In the present study, the choice of Φ_PSII_ as the main parameter characterizing photosynthetic activity was based on two factors. Firstly, Φ_PSII_ is an indicator of the efficiency of absorbed light energy use for the photosynthetic process; although it does not directly estimate the photosynthetic intensity, it shows a high correlation with CO_2_ assimilation [45]. Secondly, as mentioned above, Φ_PSII_ as a parameter characterizing the rate of electron transport in PETC is one of the most stress-sensitive parameters responding in the early stages of drought stress and at moderate stress intensity.

Analysis of the dynamics of photosynthetic activity of individual genotypes during the progression of drought stress allowed us to identify four patterns of photosynthetic activity changes, according to which the studied genotypes were divided into four groups: a group with low photosynthetic tolerance (NT), a group with tolerance of PSA to moderate short-term drought stress (MT), a group with high photosynthetic tolerance to high-intensity drought stress due to early decrease in the rate of PETC (HT), and a group of genotypes maintaining high rate of photosynthetic reactions under both moderate and more intense drought (HHT). The identified patterns probably reflect different strategies of plant behavior under drought stress.

Drought stress tolerance is a complex parameter dependent on a number of crop traits [46,47]. In wheat, most of the stress tolerance traits are polygenic and hence difficult to understand at physiological and molecular levels [48]. Currently, no single trait can be used to fully and accurately assess drought tolerance in wheat [47]. In this case, an approach based on combined indicators is used for this purpose [10,47].

Examples of different response strategies of wheat photosynthetic activity to drought have been described in the literature [49,50,51,52,53,54,55,56]. In particular, two drought-tolerant genotypes (Drysdale and Hollis) performed different drought response strategies [54]. Drysdale relies more on NPQ mechanisms to maintain high photosynthetic rates under drought conditions; Hollis is more efficient in combating stress-related ROS and oxidative damage. Under progressive drought, ChlF parameters F_v_/F_m_ and Φ_PSII_ decreased in Hollis, but were not significantly affected in Drysdale. Also, NPQ values were significantly lower throughout the drought period in the Hollis genotype. Analysis of two NPQ components, high-energy quenching (qE) and photoinhibitory-dependent quenching (qI), showed similar qE values for both genotypes, but significantly higher qI values in Drysdale. Another example of different strategies is shown by the cultivars Othalom and Kobomugi: Kobomugi retains water in the tissues by rapid closure of the stomata, maintaining osmotic potential and photosynthetic activity of the leaves, while Othalom starts to recover turgor after a relatively long phase of intense water loss due to late closure of the stomata in response to drought [56]. A comparison of the photosynthetic activity of the tall cultivar Slomer and the modern high-yielding semi-dwarf cultivar Enola showed that the higher chlorophyll content of Slomer does not fully ensure high photosynthetic efficiency. In contrast, the reduced chlorophyll content of Enola probably represents an efficient strategy to protect photosynthesis from photoinhibition and oxidative damage under stress conditions. This contributes to avoiding excessive light energy absorption and increasing the quantum efficiency of PSII, thereby maintaining an optimal photosynthetic rate [53].

Among the causes of differences in the dynamics of changes in photosynthesis activity during the drought development, differences in the level of inactivation and damage of various cell components involved in the photosynthesis process can be assumed. They can be caused by genetic features of antioxidant and hormonal systems functioning, as well as by the rate of development and intensity of different NPQ components. Rapid damage to photosystems may be caused by excessive production of ROS [15]. NPQ, as a mechanism to protect the PETC from photodamage, can suppress photosynthetic activity and simultaneously reduce the excess load on photosystems under drought stress [11,57]. In addition, the production of stress phytohormones, especially abscisic acid, is one of the key mechanisms regulating the functioning of stomata, the aperture of which affects both water loss by transpiration and CO_2_ availability, directly affecting photosynthetic activity [58].

The study of the specificity of photosynthetic response of different wheat genotypes to drought stress is an important task in the development of new tolerant cultivars with optimal behavioral strategies for their successful cultivation in specific regions. It is worth noting that photosynthetic activity studies based on gas exchange or ChlF parameters recording have limitations for the mass screening of a large number of genotypes, especially under field conditions [19,59]. A more high-throughput method for assessing plant morphophysiological traits is spectral imaging [20,60].

### 3.2. Relationship Between Photosynthetic Activity Parameters and NDIs

Soil drought induced changes in the reflectance parameters of wheat seedlings, including absolute values of reflectance and NDIs calculated in the wavelength range from 400 to 1000 nm; these changes increased with the duration of stress exposure. The greatest alterations in the reflectance coefficients were observed in the 550–700 nm and 730–1000 nm ranges. The qualitative and quantitative characteristics of the reflectance spectrum in the first range are due to the optical properties of pigments, primarily chlorophyll [20,61], which indicates changes in the pigment composition of the studied plants as a result of drought stress. Reflectance in the second most sensitive range of the spectrum shown in this work is characterized by a dependence on the structural characteristics of leaf tissues, in particular the size of the cells and intercellular spaces in the mesophyll [62]. The drought-induced shifts in the shape of wheat reflectance spectra revealed in the present work are consistent with data from other studies [63,64] and confirm the effect of drought stress on the spectral parameters of green plant parts.

NDIs also showed sensitivity to water deficit: statistically significant changes in some areas in VIS and NIR ranges were detected already three days after irrigation was stopped. With increasing duration of stress, the drought-sensitive regions in the heat maps of NDIs expanded and covered almost the whole range under study seven days after stopping irrigation. The most sensitive regions identified in our work include indices described in the literature as promising for the detection of drought stress in wheat. In particular, the sensitive regions identified at the fifth day of drought included wavelength ranges that are used to calculate a number of reflectance indices for which applicability to early detection of drought stress in wheat has been shown: Photochemical Reflectance Index (PRI = (*R*_31_ − *R*_570_)/(*R*_531_ + *R*_570_)), Photosynthetic Vigor Ratio (PVR = (*R*_550_ − *R*_650_)/(*R*_550_ + *R*_650_)), Greenness Index (G = *R*_554_/*R*_677_) [26,37]. Indices detecting more long-term drought in other papers (Lichtenthaler Index 1 (LIC1 = (*R*_800_ − *R*_680_)/(*R*_800_ + *R*_680_)), Renormalized Difference Vegetation Index (RDVI = (*R*_800_ − *R*_670_)/(*R*_800_ + *R*_670_)), Normalized Difference Vegetation Index NDVI = (*R*_780_ − *R*_670_)/(*R*_780_ + *R*_670_), Water Index (WI = *R_900_/R_970_*), Plant Pigment Ratio (PPR = (*R*_550_ − *R*_450_)/(*R*_550_ + *R*_450_)) [26,27] also showed early changes (5 days after stopping irrigation) in response to water deficit in the present study.

Seven days after irrigation stopped, the sensitive zones expanded into the wavelength ranges used to calculate the following indices: Carter Index 1 (CTR1 = R_695_/R_420_), Normalized Pigment Chlorophyll Index (NPCI = (R_680_ − R_430_)/(R_680_ + R_430_)), Red-edge Normalized Difference Vegetation Index (RNDVI = (*R*_750_ − *R*_705_)/(*R*_750_ + *R*_705_)), Zarco-Tejada and Miller (ZM = *R*_750_/*R*_710_), Vogelmann Red Edge Index 1 (VOG1 = R_740_/R_720_), NIR Shoulder Region Spectral Ratio Index (NSRI = R_890_/R_780_), Simple Ratio Pigment Index (SRPI = R_430_/R_680_), Gitelson–Merzlak Index 1 (GM1 = R_750_/R_550_), and Normalized Phaeophytinization Index (NPQI = (*R*_415_ − *R*_435_)/(*R*_415_ + *R*_435_)). These indices showed a relationship with plant or soil water content, and allowed detection of the effects of water deficit in wheat plants in previous works [26,65,66,67].

All four NDIs analyzed in this study showed a strong relationship with photosynthetic activity estimated by the Φ_PSII_ value. In addition, the groups of cultivars composed on the basis of the patterns of drought-induced Φ_PSII_ changes showed similar patterns of changes of the selected indices, which suggests the good applicability of these indices for estimating photosynthetic activity parameters under drought stress. Among the four proposed indices, NDI_572/545_ and NDI_820/630_ combined high correlation with Φ_PSII_ under drought and high sensitivity to stress: most of the cultivars showed statistically significant changes in these indices 3–5 days after stopping irrigation.

NDI_610/450_ is close to the index of PPR (550/450 nm). This index reflects pigment composition [68] and shows a relationship with the water status of the plant [26]. This relationship may be explained by the fact that chlorophylls and carotenoids show absorption peaks around 450 nm and changes in their concentration affect the intensity of reflectance in this region [69]. In the present study, the index NDI_610/450_ showed changes at drought stress duration comparable to the duration required to detect F_v_/F_m_ and Φ_NO_ shifts (10 days after stopping irrigation), which indirectly confirmed the association of changes in this index with drought-induced alterations in the pigment composition of wheat leaves.

NDI_572/545_ lies within the range of wavelengths used to calculate the normalized difference index PRI. High sensitivity to water stress as well as relationship with plant water content is shown for this index [37]. PRI reflects conversions in the violaxanthin cycle and is related to NPQ and photosynthetic efficiency [70]. It can also vary as a result of changes in leaf chlorophyll content [71]. The NDI_572/545_ used in the present work showed an increase, which corresponds to its decrease in the case if the wavelengths for the calculation were used in analogy with the PRI: λ_1_ 545 nm, λ_2_ 572 nm. In [37], the modified PRI calculated using wavelengths λ_1_ 545 nm, λ_2_ 570 nm declined in response to acute short-term drought in wheat seedlings, which is consistent with our results. The authors attributed this effect to fast-relaxing changes in PRI, which are probably related to alterations in light scattering in this range caused by chloroplast shrinkage. In our work, NDI_572/545_ detected drought stress at early stages of its development (starting 3–5 days after stopping irrigation for different genotypes) simultaneously with detecting changes in ChlF parameters Φ_PSII_ and NPQ.

NDI_740/700_ is calculated using wavelengths close to the simple ratios ZM (750/710) and VOG1 (740/720), and to the normalized difference index RENDVI (750/705). In the literature, they are shown to be related to the water content in shoots, as well as to chlorophyll content [26,72]. Changes in these indices, as in the case of the NDI_740/700_ we studied, are associated with changes in reflectance in the edge–edge band, which is a very sensitive range to stress [63,64] and reflects the chlorophyll content in the plant [73,74]. Similar to NDI_610/450_, NDI_740/700_ showed pronounced changes 10 days after stopping the irrigation, which is comparable to the timing of drought-induced changes in ChlF parameters reflecting the structural and functional integrity of photosystems (F_v_/F_m_ and Φ_NO_).

The fourth promising index NDI_820/630_ lies in the region of wavelengths used to find LIC1 (800/680), RDVI (800/670), and NDVI (780/670). All these indices have shown potential for drought detection and, in the case of NDVI, a relation to the water status of the plant [26,75]. In our work, NDI_820/630_ decreased in response to drought, which is consistent with the directionality of changes in the mentioned indices. Along with NDI_572/545_, NDI_820/630_ showed statistically significant changes earlier than the indices NDI_610/450_ and NDI_740/700_ (5 days after stopping irrigation), which, together with the high correlation between this index and Φ_PSII_ under drought, makes it promising for use in screening genotypes for drought tolerance and the sensitivity of photosynthesis to water deficit.

At the present time, the idea of using spectral imaging techniques to estimate parameters related to photosynthesis activity in plants is widespread [76]. This is primarily based on the fact that spectral imaging methods have higher throughput than techniques for recording ChlF parameters [59,77,78]. This fact, together with the good agreement between NDIs and PAM parameters shown in this work, argues in favor of the promising potential of an approach based on the use of specific reflectance indices to estimate photosynthetic activity parameters when investigating the response of genotypes to drought stress during the breeding process.

## 4. Materials and Methods

### 4.1. Plant Material

The seedlings of twenty four cultivars of soft spring wheat (*Triticum aestivum* L.) were used in the research: Temp, Ekada 279, Karavayka, Modava, Pamyati Tyunina, L-III, SU Akhab, Traditsiya, Blesk, Zagadka, Ul’yanovskaya 105, Nitsa, Vestochka 17, Madam, Znamya, Katun’, Sviyaga, Iren’, Zlata, Happy, Zaural’skaya volna, Zaural’skiy yantar’, Astrid, Sudarynya (hereinafter, cultivars C1–C24, respectively). The genetic diversity of the cultivars used was expected to provide variability in the responses of plants of different genotypes to drought.

### 4.2. Experiment Design

Plants were grown in pots (7 cm × 7 cm × 7 cm (343 mL), 5 plants per pot) with commercial peat soil (Peter Peat Agro Black, Peter Peat, Moscow, Russia), where levels of NPK were 100 mg/l N, 80 mg/l P, 130 mg/l K, and pH was at least 5.5. A pot experiment was carried out under controlled conditions in a vegetation room at air temperature 24 °C, relative humidity of about 50%, and a photoperiod of 16/8 (light/dark) with LED light (LED Cool White/6500 K, 50 W, IEK, Moscow, Russia) with 220 μmol m^−2^ s^−1^ light intensity.

**Figure 12:**
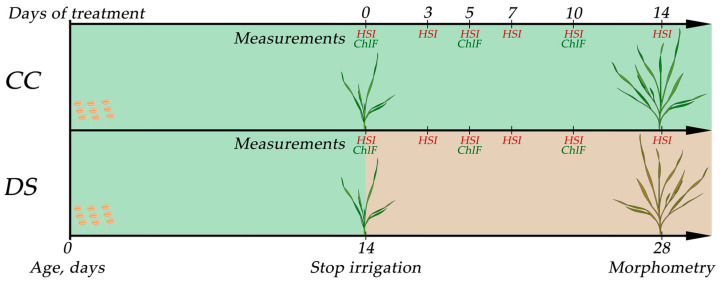
Experiment design. CC—control group, DS—drought stress treatment, HSI—hyperspectral imaging, ChlF—chlorophyll fluorescence imaging.

Plants of each cultivar were divided into two groups. In the control group (CC), plants were irrigated every 2 days throughout the entire experiment to maintain soil moisture at least 70%, calculated as:

$${\mathrm{WC}}_{\mathrm{soil}}=\frac{\mathrm{FW}-\mathrm{DW}}{\mathrm{FW}}*100\%;$$

in the drought-stressed group (DS), the irrigation of plants was stopped at the age of 14 days to create the soil drought conditions. The measurements of the reflectance parameters were conducted at the age of 14, 17, 19, 21, 24, and 28 days (before DS and after 3, 5, 7, 10, and 14 days of DS) for all experimental groups. The ChlF parameters were recorded at the age of 14, 19, and 24 days (before DS and after 5 and 10 days of DS) (Figure 12). Morphometric parameters were evaluated at the age of 28 days.

### 4.3. Morphometric Parameters

Fresh (FW) and dry (DW) weight of wheat plants were evaluated at the age of 28 days. Wheat plant were weighed using an analytical balance (Adventurer AX324, Ohaus, Parsippany, NJ, USA), then placed in the drying oven for 4 h at 100 °C; DW of the plants was then determined using the analytical balance.

The water content (WC) in wheat plants was calculated as:

$${\mathrm{WC}}_{\mathrm{plant}}=\frac{\mathrm{FW}-\mathrm{DW}}{\mathrm{FW}}*100\%.$$

### 4.4. ChlF Parameters

Photosynthetic activity parameters were recorded using a ChlF-imaging system based on pulse-amplitude modulation (PAM) technology (Plant Explorer PRO+, PhenoVation, Netherlands). The dark ($F_{0}$) and maximum ($F_{m}$) fluorescence yields in were measured after 10 min of dark adaptation. Actinic light (AL, cool white light, 200 μmol m^−2^ s^−1^) was then switched on. The current ($F_{t}$) fluorescence yield and the maximum fluorescence yield $\left(F_{m}^{\prime }\right)$ in the light-adapted state were determined 10 min after AL was switched on. The maximum fluorescence yields in the dark- and light-adapted state ($F_{m}$ and $F_{m}^{\prime }$, respectively) were measured using a saturating pulse (cool white light, 4000 μmol m^−2^ s^−1^, 800 ms duration, 6500 K).

The recorded values were used to calculate the following parameters of photosynthetic activity: the maximum quantum efficiency of PSII ($F_{v}/F_{m})$, the effective photochemical quantum yield of PSII (Φ_PSII_), the quantum yield of regulated non-photochemical energy dissipation in PSII (Φ_NPQ_), and the quantum yield of non-regulated non-photochemical energy loss in PSII (Φ_NO_). The values of $F_{v}/F_{m}$, Φ_PSII_, Φ_NPQ_, and Φ_NO_ were calculated by the software integrated into the registration system using the equations:

$$F_{v}/F_{m}=\frac{\left(F_{m}-F_{0}\right)}{F_{m}},$$

$$\Phi_{\mathrm{PSII}}=\frac{\left(F_{m}^{\prime }-F_{t}\right)}{F_{m}^{\prime }},$$

$$\Phi_{\mathrm{NPQ}}=\frac{F}{F_{m}^{\prime }}-\frac{F}{F_{m}},$$

$$\Phi_{\mathrm{NO}}=\frac{F_{t}}{F_{m}},$$

where $F_{0}$ is the minimum ChlF level after dark adaptation, $F_{m}$ is the maximum ChlF yield after dark adaptation, $F_{m}^{\prime }$ is the maximum ChlF yield in the light-adapted state, and $F_{t}$ is the current ChlF level under the light [45,79].

To evaluate the effect of DS on photosynthetic activity of wheat plants, the residual values of the parameters $F_{v}/F_{m}$, Φ_PSII_, Φ_NPQ_, and Φ_NO_ were calculated as a percentage of the control [35]:

$${\mathrm{Parameter}}_{\mathrm{resid}}=\frac{{\mathrm{Parameter}}_{\mathrm{drought}}}{{\mathrm{Parameter}}_{\mathrm{control}}}*100\%.$$

### 4.5. Reflectance Parameters

The reflectance parameters of wheat plants were recorded using a hyperspectral camera (Specim IQ, Spectral Imaging Ltd., Oulu, Finland). Images were acquired under controlled lighting conditions using halogen light sources and then analyzed using software for hyperspectral data processing developed by the authors. To process the hyperspectral images, the background was excluded, then regions of interest (ROIs) were placed. Each ROI covered the entire aboveground part of plants of one pot (5 plants) above 3 cm from the soil. Thus, one reflectance spectrum was obtained from each ROI. The range of the analyzed reflectance spectra was from 400 to 1000 nm in 3 nm steps.

Normalized difference indices (NDIs) were calculated for each wavelength combination using the equation:

$$\mathrm{NDI}=\frac{I_{\lambda_{1}}-{\;I}_{\lambda_{2}}}{I_{\lambda_{1}}+{\;I}_{\lambda_{2}}},$$

where $I_{\lambda_{1}}$ and $I_{\lambda_{2}}$ are the values of the reflectance coefficient at the wavelengths λ_1_ and λ_2_, respectively.

To evaluate the effect of DS on the reflectance parameters, the values of NDIs were calculated as a percentage of the control:

$$\mathrm{NDI},\;\%=\frac{{\mathrm{NDI}}_{\mathrm{drought}}}{{\mathrm{NDI}}_{\mathrm{control}}}*100\%.$$

### 4.6. Statistics

GraphPad Prism 8 (GraphPad Software Inc., San Diego, CA, USA), Microsoft Excel (Microsoft Corporation, Redmond, WA, USA), and R (R Core Team (2022). R: A language and environment for statistical computing. R Foundation for Statistical Computing, Vienna, Austria. URL https://www.R-project.org/, accessed on 2 September 2024) software were used for the statistical processing of the results. The results of the analyses are presented as mean values with standard errors of the mean (SEM), averaged curves with SEM, averaged reflectance spectra, and heat maps of the difference in values between the CC and DS groups and Pearson correlation coefficients. Morphometric parameters were assessed integrally for a pot (5 plants) and calculated for an individual plant (*n* = 4 for each cultivar and treatment). The ChlF and reflectance parameters were assessed integrally for a pot (5 plants) (*n* = 4 for each cultivar and treatment). The Kolmogorov–Smirnov test was used to assess the normality of data distribution. Data were analyzed using one-way analysis of variance (ANOVA) followed by Tukey’s test. The *t*-test was used to evaluate the level of significance of differences between the parameters of the CC and DS groups. *p* < 0.05 was considered significant.

## 5. Conclusions

The present study has shown the prospects of using high-throughput spectral methods to estimate photosynthetic activity under drought. It was shown that soil drought causes a decrease in photosynthetic activity in wheat plants. Four patterns of drought-induced changes in photosynthesis were revealed depending on the time and severity of its suppression. In addition, it was shown that drought induces changes in reflectance parameters of wheat shoots, including reflectance coefficients and normalized difference indices (NDIs) calculated in the range from 400 to 1000 nm. It was found that NDIs calculated in specific wavelength ranges are related to the photosynthetic parameters during the development of drought stress and can be used to assess the photosynthetic activity of wheat plants under water deficit conditions. Among the identified promising indicators, we selected two NDIs that combined high sensitivity to soil drought at an early stage and high correlation with the intensity of photosynthetic processes.

At the same time, the issue of the physiological basis of the diagnostic potential of the proposed indices remains insufficiently investigated. Disclosure of the mechanisms underlying the relationship between the response of spectral parameters and the intensity of photosynthetic processes to drought requires a detailed analysis of other structural and functional parameters of plants that influence both spectral characteristics and photosynthetic activity.

The developed approach for identification of promising spectral indices for assessment of physiological features of plants can be included in agricultural practice and used for optimizing and accelerating large-scale screening of breeding material and assessing the specific features of the response of individual genotypes to drought development.

## Data Availability

Data are contained within the article and Supplementary Materials.

## Supplementary Materials

The following supporting information can be downloaded at: https://www.mdpi.com/article/10.3390/plants14010091/s1, Figure S1: ChlF parameters of wheat seedlings of CC (shaded bars) and DS (solid bars) groups at different days after stopping the irrigation; Figure S2: The residual values of ChlF parameters of wheat seedlings at different days after stopping the irrigation; Figure S3: Drought-induced dynamics of NDIs of DS wheat plants (in % of control).
