# Supplementary material for: Search of Reflectance Indices for Estimating Photosynthetic Activity of Wheat Plants Under Drought Stress
Source: Plants (Basel). 2024 Dec 31;14(1):91. doi: 10.3390/plants14010091 (PMC11723397; doi:10.3390/plants14010091)
Supplement: Supplementary file 1 [file plants-14-00091-s001.zip › plants-3396231-supplementary.pdf]

Supplementary material

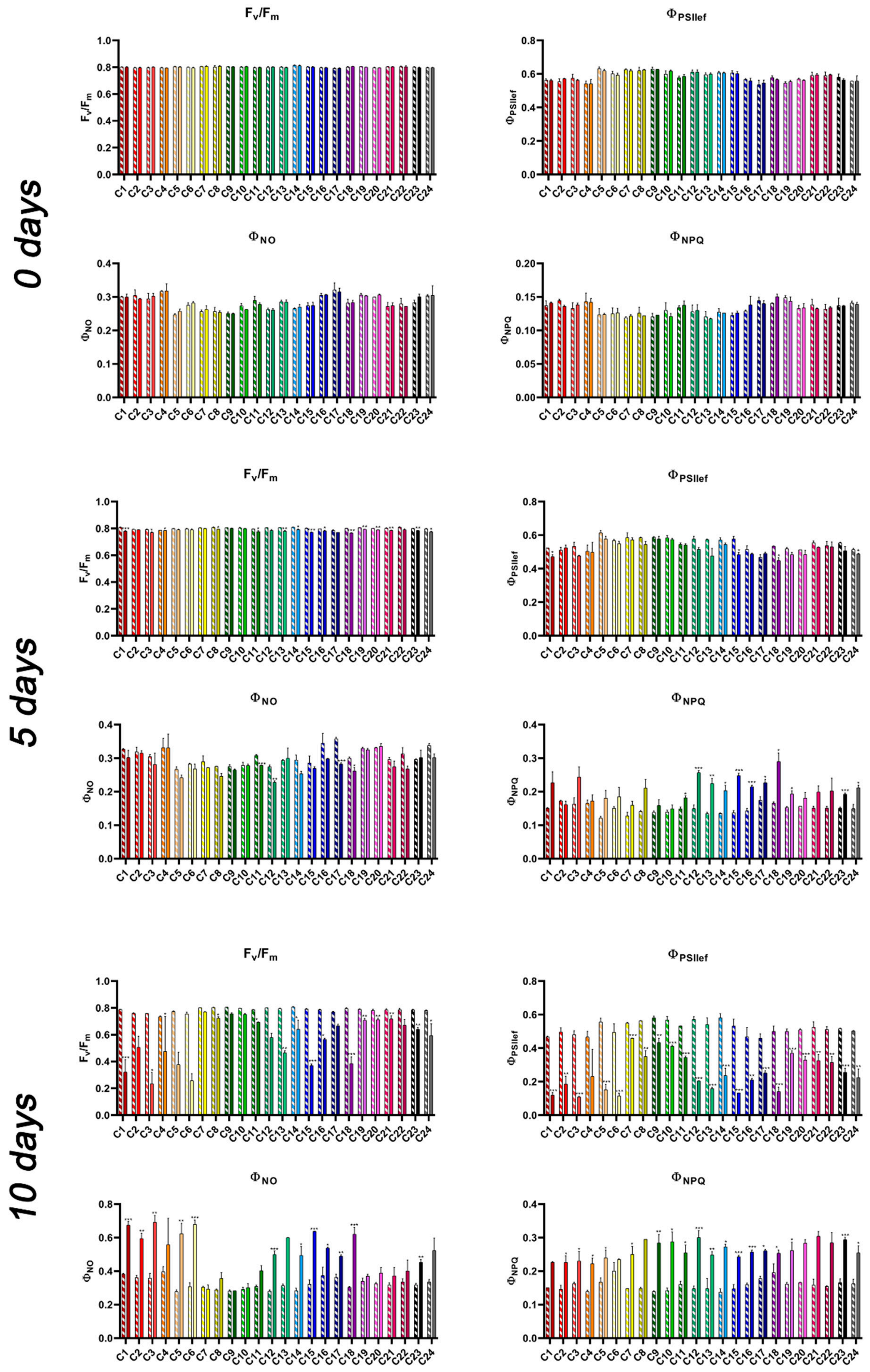

Figure S1. ChlF parameters of wheat seedlings of CC (shaded bars) and DS (solid bars) groups at different days after stopping the irrigation. Data are presented as mean ± SEM.

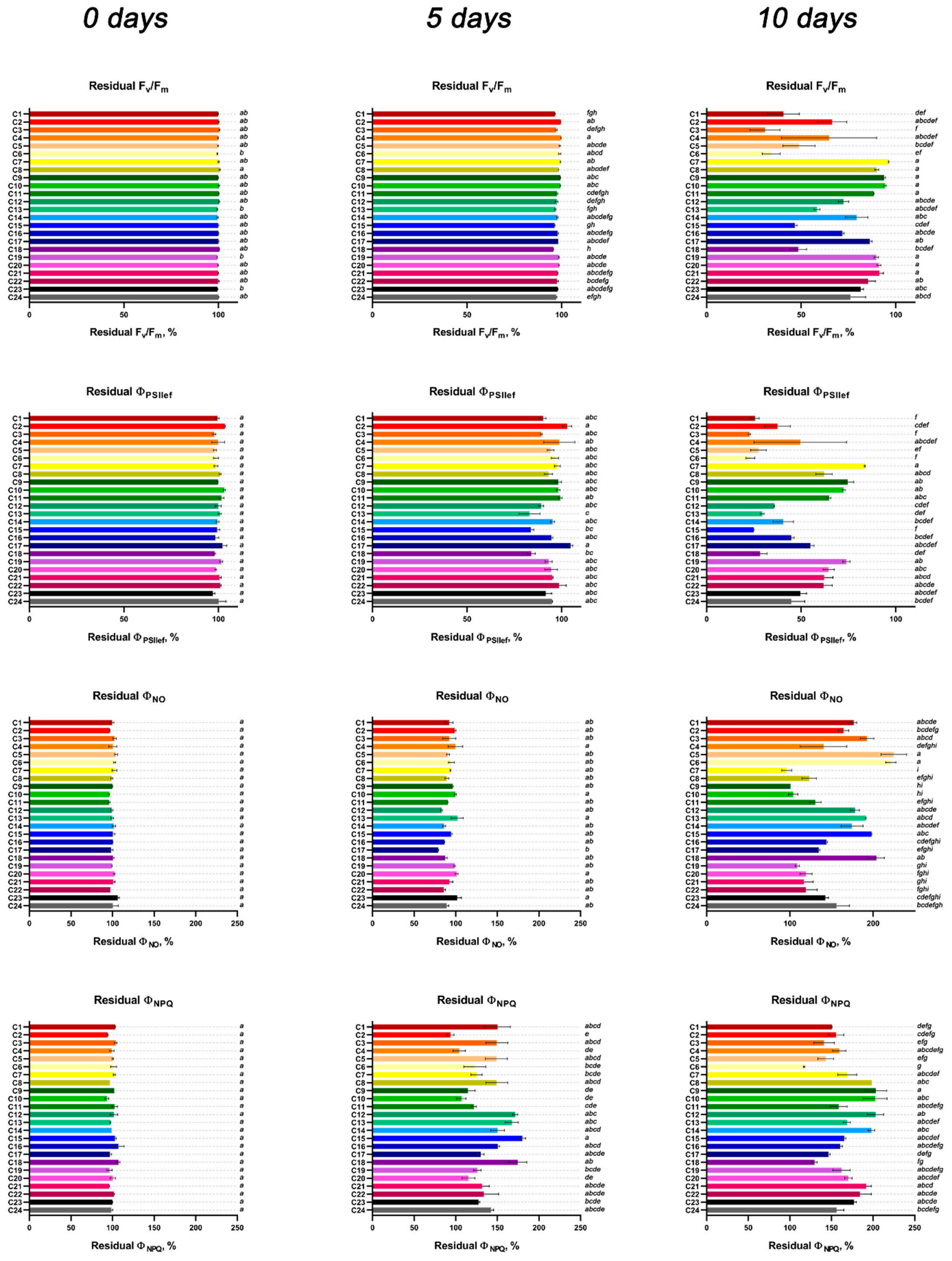

Figure S2. The residual values of ChlF parameters of wheat seedlings at different days after stopping the irrigation. Data are presented as mean ± SEM.

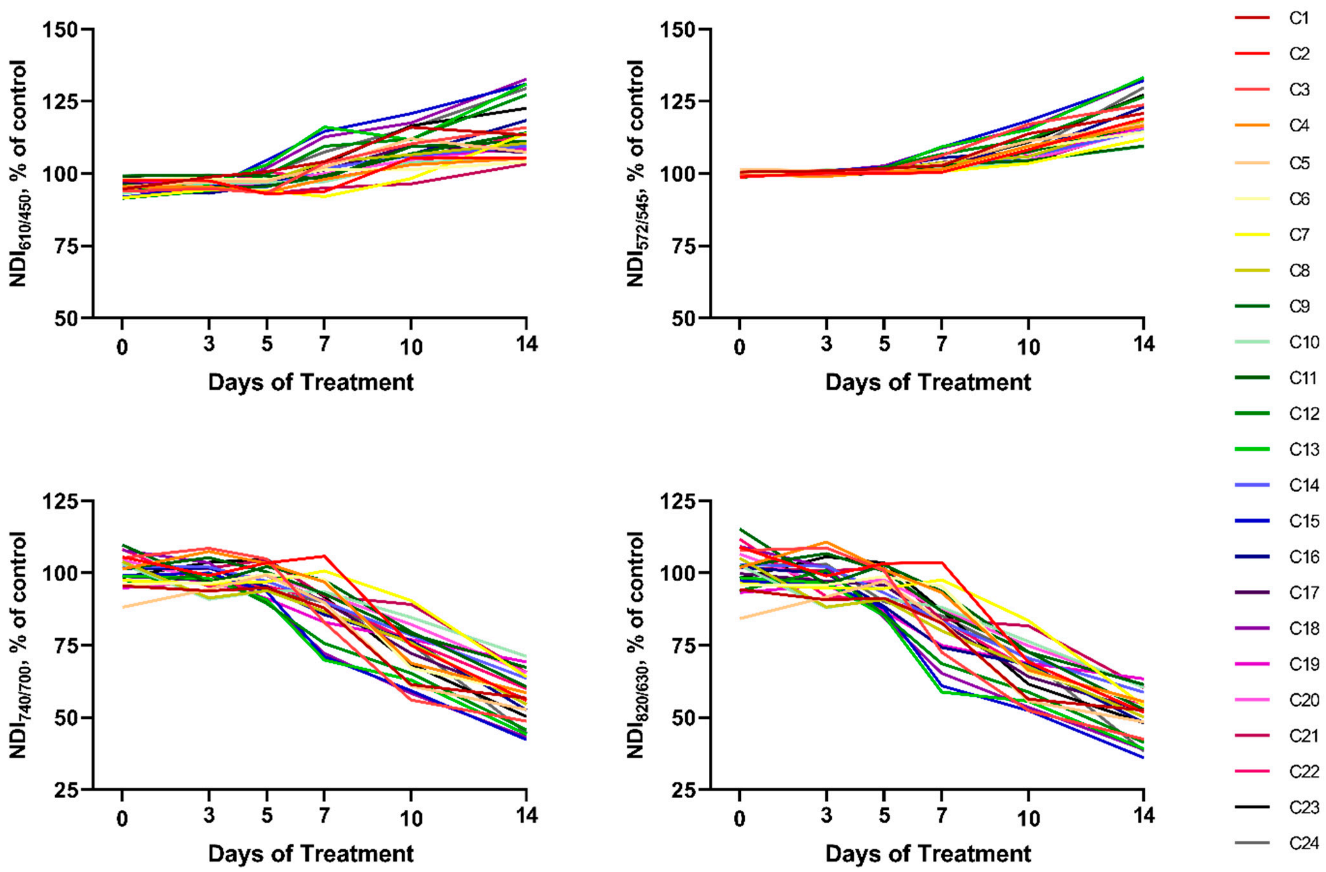

Figure S3. Drought-induced dynamics of NDIs of DS wheat plants (in % of control). Data are presented as means for each cultivar.
